# Partial Label Learning for Bearing Fault Diagnosis via Residual Shrinkage and ETF-Guided Prototype Learning

**DOI:** 10.3390/s26216738

**Published:** 2026-10-24

**Authors:** Shuai Zhang, Yubo Wang, Yanjie Pei, Mengqiang Feng, Tianci Zhao, Zhiyou Cheng

**Affiliations:** 1 School of Electronics and Information Engineering, Anhui University, Hefei 230601, China; p24101008@stu.ahu.edu.cn; 2 School of Internet, Anhui University, Hefei 230039, China

**Keywords:** bearing fault diagnosis, partial label learning, residual shrinkage, equiangular tight frame, prototype learning, noise robustness

## Abstract

Accuratebearing fault diagnosis is challenged by noise-contaminated vibration signals and ambiguous fault annotations. To address these challenges, this paper proposes RS-ETFPLL, a residual shrinkage and equiangular tight frame (ETF)-guided partial label learning (PLL) framework. Vibration signals are transformed into time–frequency representations using the continuous wavelet transform, and an adaptive residual shrinkage encoder is employed for noise-robust feature extraction. Fixed simplex ETF prototypes provide a stable and uniformly separated class geometry. Both the classification and prototype branches are trained with candidate-set supervision, while pseudo-soft labels obtained by normalizing classification predictions within candidate sets guide prototype learning to facilitate label disambiguation. Experiments on the CWRU, MFPT, and PU datasets demonstrate that RS-ETFPLL outperforms six representative PLL baselines across the evaluated ambiguity levels. In the main comparison at an incorrect-label inclusion probability of q=0.5, it achieves mean accuracies of 99.59%, 98.99%, and 98.01%, surpassing the best baseline in each setting by 2.03, 4.46, and 2.16 percentage points, respectively. At the same ambiguity level and a signal-to-noise ratio of −2 dB, the corresponding accuracies are 81.72%, 82.08%, and 73.78%. These results demonstrate the effectiveness of RS-ETFPLL for bearing fault diagnosis under the evaluated noise and candidate-label ambiguity conditions.

## 1. Introduction

Rolling bearings are essential components of rotating machinery, and their health condition directly affects the reliability and safety of mechanical systems. Prolonged operation at high speeds and under heavy loads can cause localized defects in the inner race, outer race, or rolling elements. If left undetected, these defects may develop into more severe faults, leading to performance degradation or unexpected equipment shutdown. Therefore, vibration-based bearing fault diagnosis remains an important topic in machinery condition monitoring and intelligent maintenance [1,2,3]. Deep-learning-based methods, particularly convolutional and residual networks, have been widely used to learn hierarchical fault features from raw vibration signals or their time–frequency representations, achieving promising performance on benchmark bearing datasets [4,5,6,7]. However, many of these methods are developed for fully supervised learning and require substantial amounts of training data with a single accurate label for each sample. Obtaining such annotations can be difficult in practical industrial applications.

Beyond conventional supervised fault diagnosis, recent studies have explored different strategies to address data scarcity and variations in operating conditions. Wu et al. proposed a conditional distribution-guided adversarial transfer learning network (CDGATLN) to facilitate multi-source knowledge transfer for cross-machine bearing fault diagnosis through conditional distribution alignment [8]. Wang et al. developed a dynamic collaborative adversarial domain adaptation network (DCADAN) to improve unsupervised cross-domain fault diagnosis through dynamically coordinated feature learning and domain adaptation [9]. To alleviate data scarcity, Wang et al. introduced an adaptive fused domain-cycling variational generative adversarial network (AFDVGAN), which integrates multi-domain data synthesis with adaptive fusion of synthetic and real samples [10]. Furthermore, Wang et al. proposed a related task-aware curriculum meta-learning (RT-ACM) framework that exploits auxiliary-task relevance and curriculum-based task scheduling to improve bearing fault diagnosis with limited labeled data under varying operating conditions [11]. Although these methods have advanced fault diagnosis under limited data or changing operating conditions, they do not directly address ambiguity among multiple candidate labels associated with individual training samples. Such ambiguity represents a distinct challenge that motivates the investigation of partial label learning.

Accurate fault identification often relies on expert knowledge, inspection findings, maintenance records, and other auxiliary information. When different fault categories exhibit similar vibration patterns or the available evidence is incomplete, annotators may be unable to assign a single reliable label and may instead provide several plausible candidates. Discarding these ambiguously labeled samples may result in the loss of useful training information, whereas assigning a single label without sufficient evidence risks introducing incorrect supervision. Partial label learning (PLL) offers a weakly supervised framework for exploiting such data [12,13]. In the standard PLL setting, each training instance is associated with a candidate label set that is assumed to contain its single ground-truth label, whose identity is unknown to the learner. The objective is to learn an accurate classifier while resolving the ambiguity among candidate labels [13,14,15]. PLL has also been investigated for bearing fault diagnosis; for example, Zhang et al. combined multi-center prototypes with entropy-weighted learning to address ambiguous fault annotations [12].

Existing PLL methods address candidate-label ambiguity through prediction-based confidence refinement, theoretically grounded learning objectives, and discriminative representation learning. PRODEN progressively updates candidate-label confidence using model predictions, jointly optimizing the classifier and identifying the latent true labels [16]. Feng et al. developed a model of candidate-label generation and proposed two PLL approaches, namely risk-consistent (RC) and classifier-consistent (CC) learning, with consistency guarantees under the corresponding generation assumptions [17].

Other studies explicitly exploit the structure of the learned feature space to support label disambiguation. PiCO combines contrastive representation learning with prototype-based label disambiguation, allowing feature learning and candidate-label refinement to reinforce each other [18]. ParSE incorporates semantic label representations to facilitate representation learning and candidate-label disambiguation [19]. PaPi uses a linear classifier to guide the optimization of a prototypical classifier sharing the same feature encoder, thereby improving representations for partial-label classification [20]. Together, these studies highlight the importance of coupling label disambiguation with discriminative feature learning and class-level structural information.

Despite these advances, reliable bearing fault diagnosis under partial-label supervision still faces two related challenges. First, measurement noise and environmental disturbances can obscure fault-related vibration patterns, reducing the reliability of the features and predictions used for candidate-label disambiguation. Residual shrinkage networks incorporate adaptive soft-thresholding into feature extraction to suppress noise interference [21,22,23,24]. This motivates integrating adaptive shrinkage into PLL to improve the fault representations on which label disambiguation depends.

Second, prototype-based PLL methods provide useful class-level structural information [25], but prototypes estimated from uncertain predictions may be affected by incorrect candidate-label assignments. Prediction uncertainty can therefore affect not only individual label estimates but also the class-level references used to guide feature learning. Studies on neural collapse have shown that normalized, globally centered class-mean feature vectors can approach a simplex equiangular tight frame (ETF) during the terminal phase of supervised training under suitable conditions [26]. Related work has explored fixed ETF classifiers to induce this geometry [27], with further applications in federated and semi-supervised learning [28,29]. These findings motivate using fixed ETF prototypes with equal pairwise angular separation as stable class references. Candidate-label supervision guides the association between the learned fault features and these fixed references.

To address these challenges, this paper proposes a Residual Shrinkage and ETF-Guided Partial Label Learning framework, termed RS-ETFPLL, for bearing fault diagnosis. An adaptive residual shrinkage encoder extracts fault representations for a classification branch and a prototype branch guided by fixed ETF vectors. Candidate-set supervision is applied to both branches. The classification branch further generates confidence-based pseudo-soft labels by normalizing its predictions within each candidate set, providing additional guidance for the prototype branch. This joint formulation combines noise-robust feature learning with fixed geometric references and prediction-based label disambiguation.

The main contributions of this work are summarized as follows:

- A noise-robust PLL framework, RS-ETFPLL, is developed for bearing fault diagnosis. Adaptive residual shrinkage is incorporated into the encoder to suppress noise-related responses and support fault representation learning under candidate-label supervision.
- Fixed simplex ETF prototypes are incorporated into a prototype branch to provide a stable class geometry with equal pairwise angular separation. Candidate-set supervision guides feature learning within this structured space.
- A joint optimization strategy combines candidate-set supervision in both branches with a Kullback–Leibler (KL) divergence loss that aligns prototype predictions on mixed inputs with the corresponding mixed pseudo-soft labels. Experiments on the CWRU, MFPT, and PU datasets demonstrate the effectiveness of the framework under synthetically generated candidate-label ambiguity and controlled Gaussian noise.

## 2. Proposed Method

**Figure 1:**
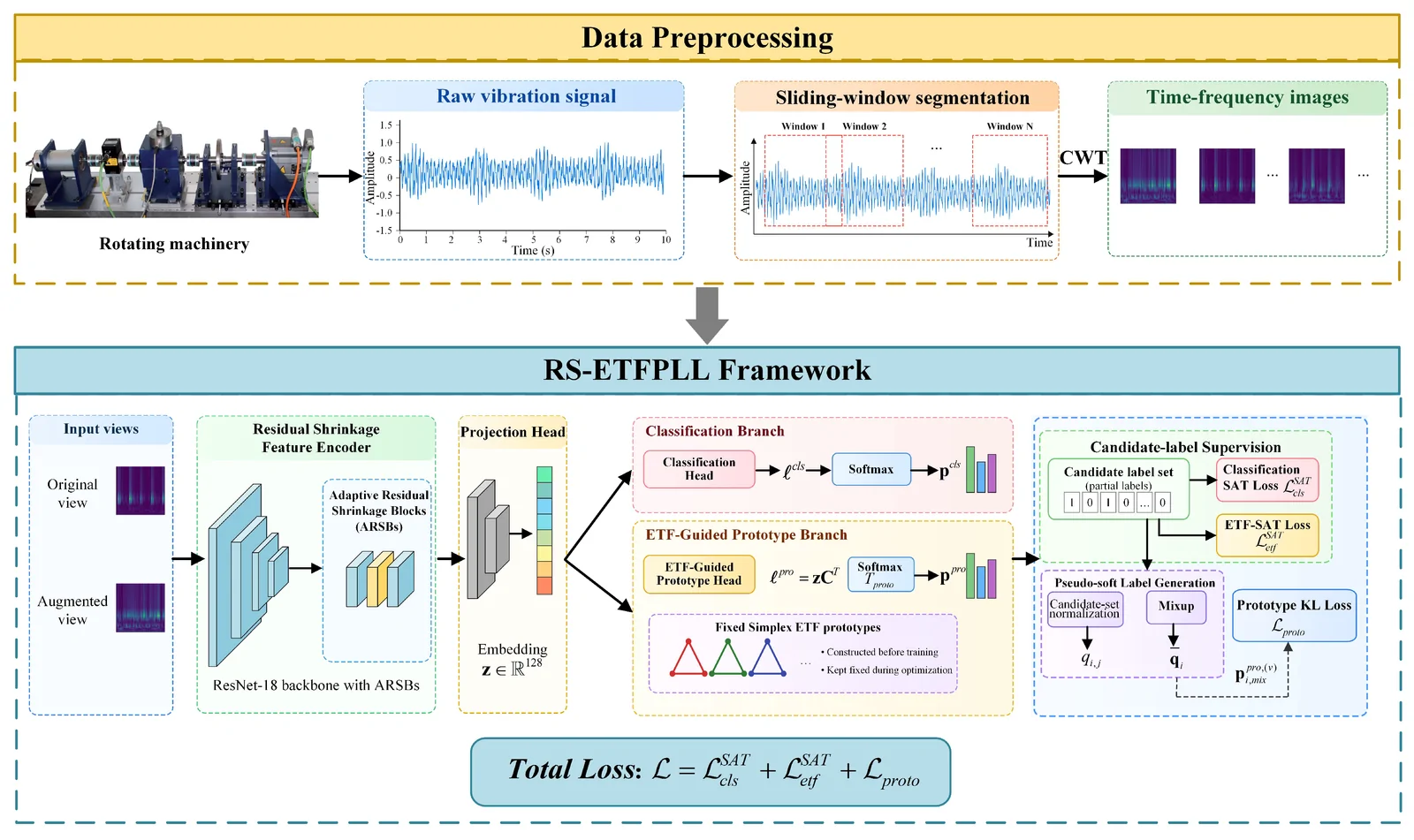
Overall framework of the proposed method.

In this section, we introduce the proposed RS-ETFPLL (Residual Shrinkage and ETF-Guided Partial Label Learning) framework for bearing fault diagnosis. First, raw vibration signals are segmented using a sliding-window strategy and transformed into two-dimensional time–frequency representations through the continuous wavelet transform (CWT). The resulting images are fed into a shared residual shrinkage feature encoder built on a modified ResNet-18 backbone, where adaptive residual shrinkage blocks are incorporated to suppress noise-related responses and extract discriminative fault features. The extracted features are then processed by two complementary branches: a classification branch and an ETF-guided prototype branch. The classification branch produces class probabilities for candidate-label disambiguation, while the prototype branch employs fixed simplex ETF prototypes to provide stable geometric references for fault representation learning. During training, both branches are optimized under candidate-set supervision, and pseudo-soft labels derived from classification predictions further guide prototype learning. By integrating noise-robust feature extraction with structured prototype learning, RS-ETFPLL aims to address noise interference and candidate-label ambiguity within a unified framework. The overall architecture is illustrated in Figure 1.

### 2.1. Partial Label Learning (PLL)

**Figure 2:**
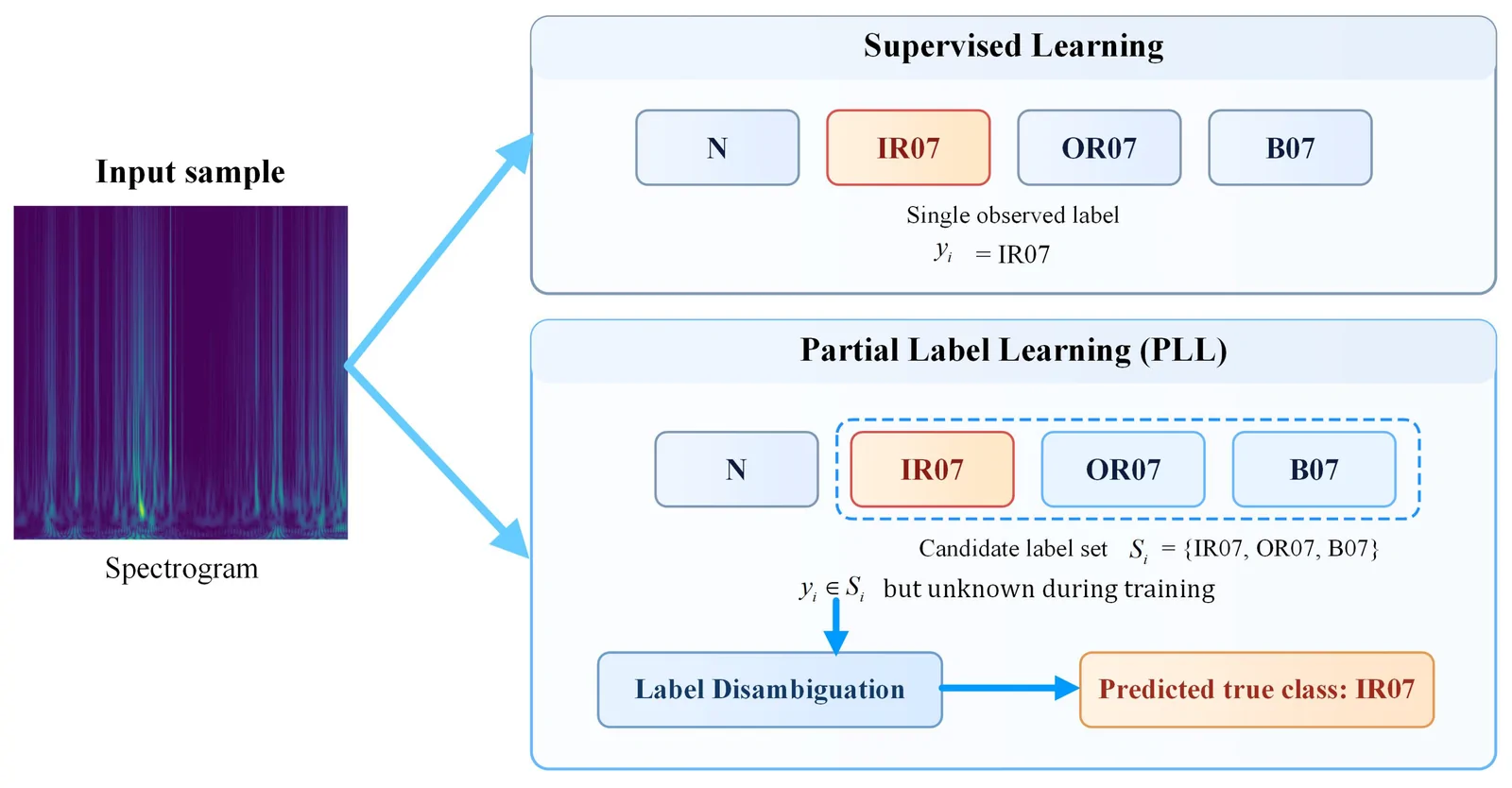
Illustration of fully supervised learning and PLL for bearing fault diagnosis. The highlighted candidate in the PLL panel indicates the ground-truth label for illustration only; its identity is not revealed to the learner during training.

Partial label learning (PLL) is a weakly supervised learning paradigm in which each training sample is associated with a set of candidate labels rather than a single class label [30,31]. The candidate label set is assumed to contain the ground-truth label, but its identity within the set is not revealed to the learner during training. As illustrated in Figure 2, fully supervised learning provides the ground-truth label for each training sample, whereas PLL provides a candidate label set from which the model seeks to identify the latent true class.

Formally, let X denote the input space and Y={1,2,…,K} denote the label space containing *K* classes. For a training sample $x_{i}\in X$ with an unobserved ground-truth label $y_{i}\in Y$, PLL provides a candidate label set $S_{i}\subseteq Y$ satisfying $y_{i}\in S_{i}$. Typically, $S_{i}$ contains the true label together with several incorrect candidate labels. Therefore, PLL aims to learn an accurate classifier while progressively resolving the ambiguity among candidate labels.

In bearing fault diagnosis, such label ambiguity may arise from similar fault characteristics or incomplete prior knowledge during annotation. Consequently, an effective PLL method should learn discriminative fault representations while suppressing the influence of incorrect candidate labels, thereby encouraging higher confidence in the true fault category during training.

### 2.2. Residual Shrinkage Feature Encoder

**Figure 3:**
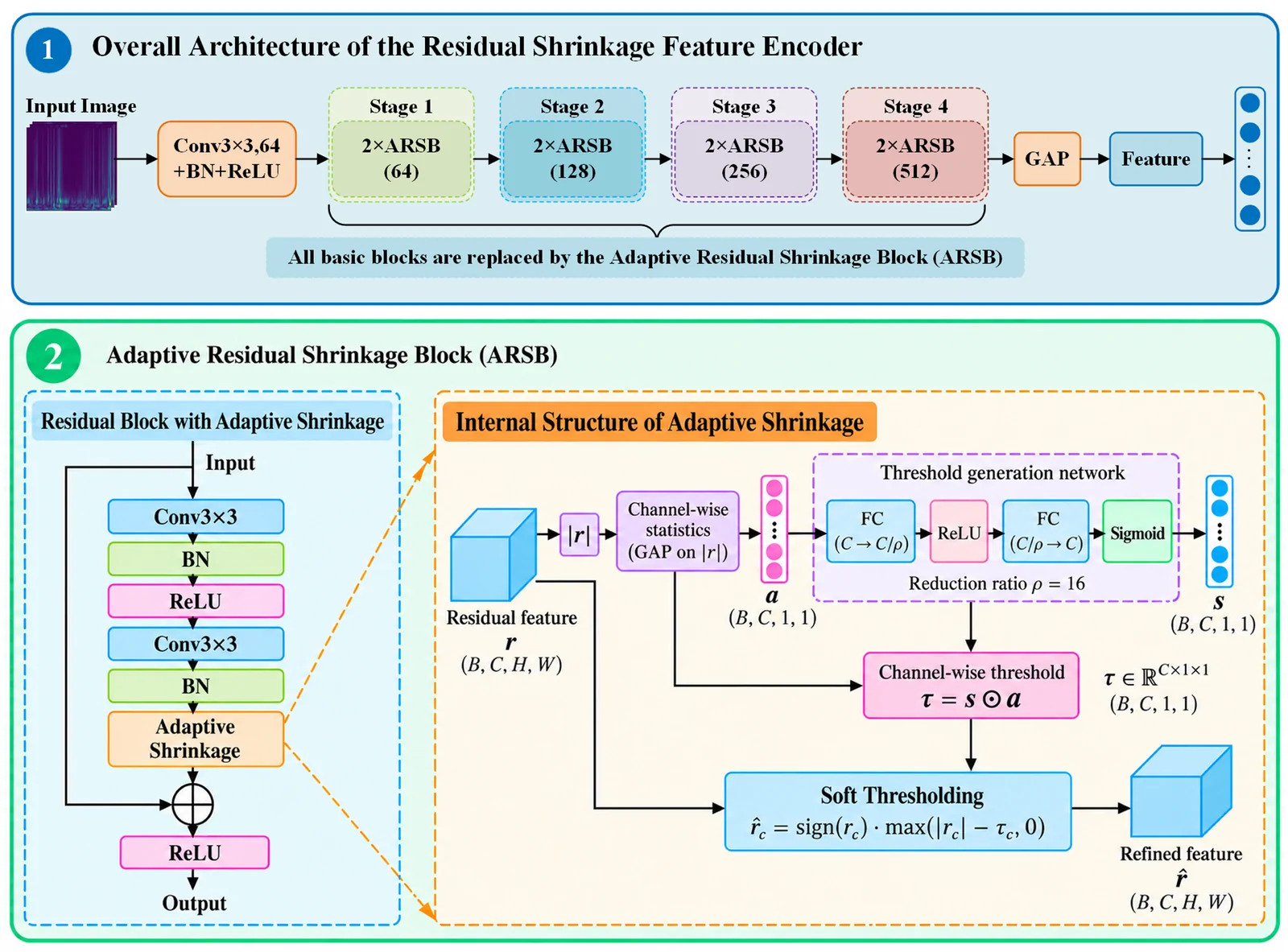
Architecture of the residual shrinkage feature encoder and the adaptive residual shrinkage block (ARSB). Here, *B* denotes the batch size.

In practical rotating machinery applications, bearing vibration signals are affected by measurement noise, environmental disturbances, and variations in operating conditions [22]. Consequently, the time–frequency representations obtained using CWT may contain responses unrelated to bearing faults. Although residual connections in ResNet-18 facilitate deep feature learning [32], its standard residual blocks do not explicitly incorporate input-dependent thresholding to suppress such responses. To address this issue, a Residual Shrinkage Feature Encoder is built on a modified ResNet-18 backbone, in which the standard basic residual blocks are replaced with Adaptive Residual Shrinkage Blocks (ARSBs), as illustrated in Figure 3. Each ARSB incorporates channel-wise adaptive soft-thresholding into the residual branch to suppress nuisance responses during feature extraction [21].

The encoder begins with a 3×3 convolutional layer followed by batch normalization (BN) and ReLU. This is followed by four residual stages, each containing two ARSBs. The four stages have 64, 128, 256, and 512 output channels, respectively. After hierarchical feature extraction, global average pooling (GAP) converts the final feature map into a 512-dimensional feature vector. A subsequent projection head maps this vector into a 128-dimensional embedding space for the classification and ETF-guided prototype branches.

For clarity, the batch dimension is omitted in the following formulation, and channel-wise quantities are written in vector form. Given an input feature map x, the residual branch of an ARSB first computes

(1) r=F(x),

where F(·) denotes the residual mapping consisting of two 3×3 convolutional layers, each followed by BN, with a ReLU activation between them. Unlike a standard residual block, an ARSB applies adaptive shrinkage to r before adding it to the shortcut output.

Specifically, GAP is applied to the absolute residual responses to obtain channel-wise magnitude statistics. For a residual feature map $r\in R^{C\times H\times W}$, the mean absolute response of the *c*-th channel is calculated as

(2) $$a_{c}=\frac{1}{HW}\sum_{h=1}^{H}\sum_{w=1}^{W}\left|r_{c,h,w}\right|,$$

where *C* denotes the number of feature channels in the current block, and *H* and *W* denote the spatial height and width, respectively. Collecting these statistics yields the channel descriptor $a\in R^{C}$.

The descriptor a is then fed into a lightweight threshold generation network consisting of two fully connected layers. The first layer is followed by ReLU, while the second is followed by a sigmoid activation to produce channel-wise scaling coefficients. These coefficients are multiplied elementwise by the magnitude statistics to obtain the adaptive thresholds:

(3) $$s=\sigma \;\left(W_{2}\delta \;\left(W_{1}a\right)\right),\;\tau =s⊙a,$$

where $W_{1}$ and $W_{2}$ are the learnable weight matrices of the two fully connected layers, δ(·) and σ(·) denote ReLU and sigmoid, respectively, and ⊙ represents elementwise multiplication. Both s and τ are *C*-dimensional vectors. The threshold generation network adopts a bottleneck structure with a channel reduction ratio of ρ=16. The sigmoid activation constrains each scaling coefficient to (0,1), while multiplication by a adapts the threshold to the mean absolute response of the corresponding channel. Thus, the thresholds depend on the current sample and feature channel.

Using the resulting thresholds, soft-thresholding is applied to the residual feature [33]. The refined feature of the *c*-th channel is expressed as

(4) $${\hat{r}}_{c}=\mathrm{sign}\left(r_{c}\right)⊙\max\;\left(|r_{c}|-\tau_{c},\;0\right),$$

where $\tau_{c}$ is the threshold for the *c*-th channel. All operations are performed elementwise, and the same threshold $\tau_{c}$ is shared across the spatial locations within that channel. This operation sets responses whose magnitudes do not exceed the threshold to zero and shrinks the remaining responses toward zero. The input-dependent thresholds therefore provide channel-wise control over feature attenuation, with the aim of suppressing noise-related and weakly informative responses.

Finally, the refined residual feature is added to the shortcut output, followed by a ReLU activation:

(5) $$y=\mathrm{ReLU}\;\left(\hat{r}+S\left(x\right)\right),$$

where S(·) denotes the shortcut mapping. An identity mapping is used when the input and output have the same spatial dimensions and number of channels; otherwise, a 1×1 convolutional projection is employed to match these dimensions.

By incorporating adaptive shrinkage into residual learning, the encoder explicitly regulates residual feature responses according to their channel-wise magnitude statistics. This design is intended to reduce the propagation of nuisance information and provide more robust fault representations for subsequent ETF-guided prototype learning and partial-label disambiguation.

### 2.3. ETF-Guided Prototype Learning

**Figure 4:**
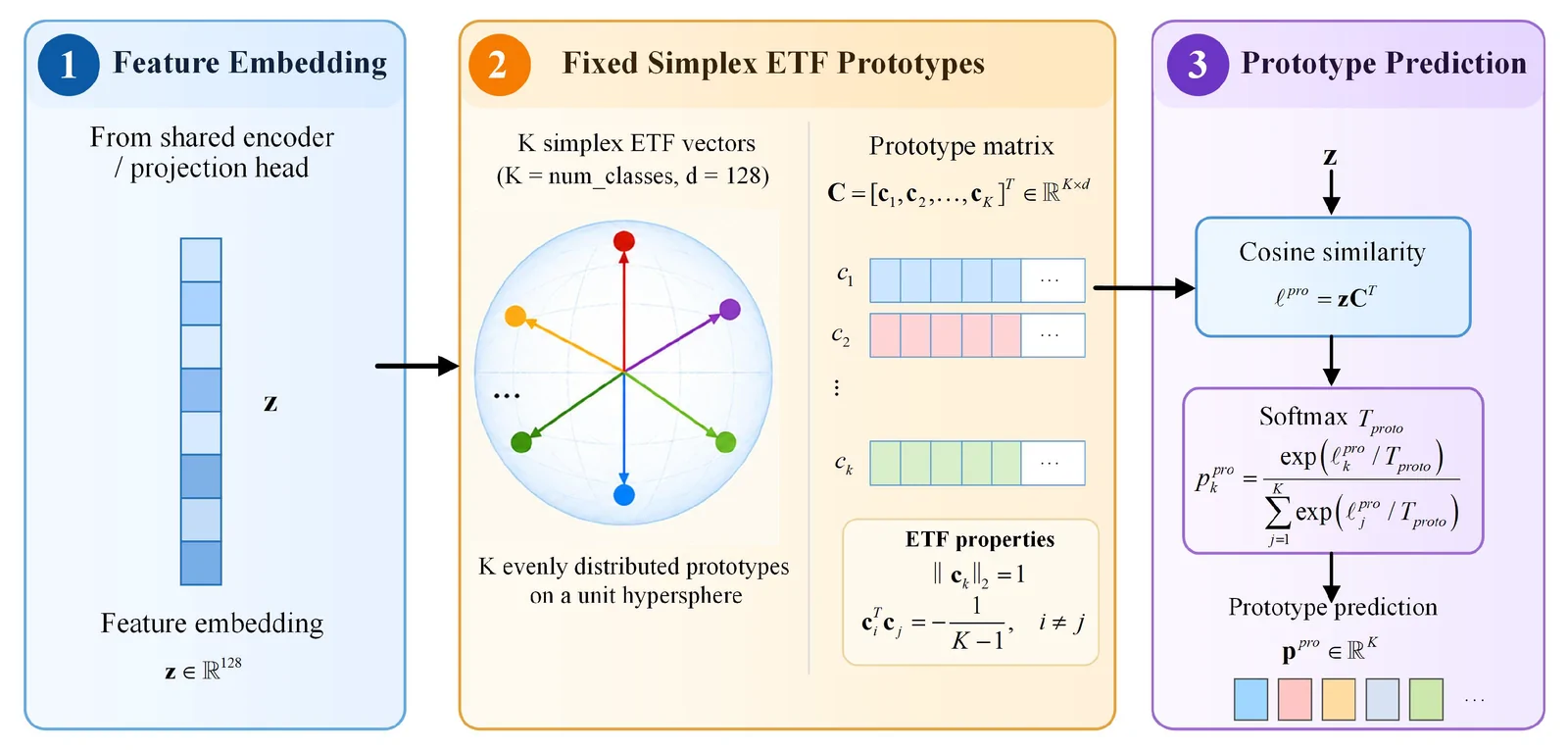
Illustration of the proposed ETF-guided prototype learning mechanism. The sphere schematically illustrates the high-dimensional prototype geometry.

In the partial label learning setting, each training sample is associated with a candidate label set, which may introduce uncertainty into the supervision. When class prototypes are estimated from uncertain classification predictions, the resulting class structure may be affected by incorrect candidate-label assignments [18,20]. To provide a stable and well-separated geometric reference for different fault categories, an ETF-guided prototype learning mechanism is introduced alongside the classification branch. Specifically, fixed simplex equiangular tight frame (ETF) vectors are employed as class prototypes [26,34], and prototype predictions are obtained by measuring the similarities between the learned feature representation and these structured prototypes. The overall procedure is illustrated in Figure 4.

After feature extraction by the Residual Shrinkage Feature Encoder, the 512-dimensional feature vector is mapped into a *d*-dimensional embedding space through a projection head. In this work, the embedding dimension is set to d=128. The projected feature is then $\ell_{2}$-normalized and denoted by $z\in R^{d}$, with ${∥z∥}_{2}=1$. For the matrix multiplication below, z is represented as a row vector.

For a fault diagnosis task with K≥2 classes, one fixed ETF prototype is assigned to each class. A simplex ETF with *K* prototypes requires an embedding dimension satisfying d≥K−1. The resulting prototype matrix is defined as

(6) $$C={\left[c_{1},c_{2},\ldots ,c_{K}\right]}^{T}\in R^{K\times d},$$

where $c_{k}\in R^{d}$ is a column vector representing the prototype associated with the *k*-th fault category. Thus, the *k*-th row of C is $c_{k}^{T}$. Unlike trainable classifier weights, the ETF prototypes are constructed before training and remain fixed throughout optimization, following the fixed-ETF approach adopted in neural-collapse-inspired learning frameworks [35]. The simplex ETF prototypes satisfy

(7) $$∥c_{k}{∥}_{2}=1,\;c_{i}^{T}c_{j}=-\frac{1}{K-1},\;i\neq j.$$

Consequently, all prototypes have unit norm and identical pairwise inner products. They lie on the unit hypersphere and form the vertices of a regular simplex, providing a symmetric class geometry with equal pairwise angular separation.

Given the normalized feature representation z, its similarities to all ETF prototypes are computed to obtain the prototype logits:

(8) $$\ell^{\mathrm{pro}}=zC^{T},$$

where $\ell^{\mathrm{pro}}\in R^{K}$ denotes the prototype logit vector, represented as a row vector in this expression. Since both z and the prototype vectors are normalized, each element of $\ell^{\mathrm{pro}}$ equals the cosine similarity between the sample feature and the corresponding class prototype.

A temperature-scaled softmax operation is subsequently applied to convert the prototype logits into class probabilities:

(9) $$p_{k}^{\mathrm{pro}}=\frac{\exp\left(\ell_{k}^{\mathrm{pro}}/T_{\mathrm{proto}}\right)}{\sum_{j=1}^{K}\exp\left(\ell_{j}^{\mathrm{pro}}/T_{\mathrm{proto}}\right)},\;k=1,\ldots ,K,$$

where $T_{\mathrm{proto}}>0$ is the temperature parameter controlling the sharpness of the predicted probability distribution. The resulting probability vector is denoted by $p^{\mathrm{pro}}\in R^{K}$.

By keeping the ETF prototypes fixed rather than estimating them from uncertain predictions, the prototype branch maintains a stable class geometry throughout training. The encoder and projection head are optimized under candidate-label supervision to learn feature representations with respect to these fixed references. This design is intended to promote discriminative fault representations and support partial-label disambiguation within the joint optimization framework. Compared with unconstrained or learnable prototypes, fixed ETF prototypes provide a predefined class geometry with equal pairwise angular separation. This property is particularly beneficial in partial-label learning, where prototype estimation may be affected by uncertain candidate-label assignments.

### 2.4. Partial-Label Supervision and Joint Optimization

RS-ETFPLL is jointly optimized using candidate-label supervision and confidence-based pseudo-soft labels derived from the classification branch. The candidate-set losses use a differentiable soft disjunction implemented by the probabilistic-sum operator [36]. The training objective combines three components: the classification SAT loss, the ETF-SAT loss, and the prototype KL loss, where SAT denotes satisfiability-based supervision.

First, the candidate label set is used to supervise the classification branch. For the *i*-th training sample, let $S_{i}$ denote its candidate label set and let $p_{i,j}^{\mathrm{cls}}$ denote the predicted probability of class *j*. Since the ground-truth label is assumed to belong to $S_{i}$, supervision is applied at the candidate-set level without explicitly selecting a single candidate as the target. The classification SAT loss is defined as

(10) $$L_{\mathrm{cls}}^{\mathrm{SAT}}=-\frac{1}{B}\sum_{i=1}^{B}\log\left[1-\prod_{j\in S_{i}}\left(1-p_{i,j}^{\mathrm{cls}}\right)\right],$$

where *B* denotes the batch size. This objective encourages a confident prediction for at least one candidate class without requiring an explicit hard-label assignment.

Second, the same candidate-set supervision is applied to the ETF-guided prototype branch. Let $p_{i,j}^{\mathrm{pro},(v)}$ denote the prototype prediction probability of class *j* for the *v*-th view of sample *i*, where v∈{1,2}. Both views correspond to the same underlying sample and share the candidate label set $S_{i}$. The ETF-SAT loss is defined as

(11) $$L_{\mathrm{etf}}^{\mathrm{SAT}}=-\frac{1}{2B}\sum_{v=1}^{2}\sum_{i=1}^{B}\log\left[1-\prod_{j\in S_{i}}\left(1-p_{i,j}^{\mathrm{pro},(v)}\right)\right].$$

This loss encourages both views to assign high probability to candidate classes using the fixed ETF prototypes as class references. The factor 1/(2B) averages the loss over the two views and the samples in the batch. The prototypes remain fixed, while the feature representations are optimized under this supervision.

Although SAT supervision encourages confident predictions within the candidate set, it does not explicitly provide a soft target distribution over the candidate classes. To obtain such targets, the classification probabilities are normalized within each candidate set:

(12) $$q_{i,j}=\frac{p_{i,j}^{\mathrm{cls}}\;I\left(j\in S_{i}\right)}{\sum_{k\in S_{i}}p_{i,k}^{\mathrm{cls}}},\;j=1,\ldots ,K,$$

where I(·) is the indicator function and *K* is the number of classes. The resulting vector $q_{i}$ assigns zero probability to non-candidate classes and sums to one over $S_{i}$. Candidate classes with higher classification probabilities therefore receive larger weights in the soft target.

To match the Mixup operation in the input space [37], the pseudo-soft labels of paired samples are combined using the same mixing coefficient:

(13) $${\bar{q}}_{i}=\lambda q_{i}+\left(1-\lambda \right)q_{\pi (i)},$$

where λ∈[0,1] is the mixing coefficient and π denotes a permutation of the batch indices. The two mixed views use the same sample pairing and mixing coefficient, allowing them to share ${\bar{q}}_{i}$ as their soft target. Thus, the mixed target combines the classification-derived confidence information of the paired samples in the same proportions as their inputs.

The mixed pseudo-soft labels are then used to guide the prototype predictions through a Kullback–Leibler (KL) divergence loss:

(14) $$L_{\mathrm{proto}}=\frac{1}{2B}\sum_{v=1}^{2}\sum_{i=1}^{B}D_{\mathrm{KL}}\left({\bar{q}}_{i}\;\|\;p_{i,\mathrm{mix}}^{\mathrm{pro},(v)}\right),$$

where $p_{i,\mathrm{mix}}^{\mathrm{pro},(v)}$ denotes the prototype prediction distribution for the *v*-th mixed view of sample *i*. This loss encourages the prototype predictions on mixed inputs to match the corresponding mixed pseudo-soft targets. Despite candidate-set normalization, dual-branch SAT supervision, and fixed ETF prototypes, prediction-derived pseudo-soft labels may still introduce confirmation bias.

Finally, the overall training objective is the sum of the three loss components:

(15) $$L=L_{\mathrm{cls}}^{\mathrm{SAT}}+L_{\mathrm{etf}}^{\mathrm{SAT}}+L_{\mathrm{proto}}.$$

The two SAT losses provide candidate-set supervision for the classification and prototype branches, respectively, while the prototype KL loss incorporates classification-derived soft targets for mixed inputs. Together, these objectives support candidate-label disambiguation and discriminative fault representation learning within a unified optimization framework.

## 3. Experiments and Analysis

This section evaluates RS-ETFPLL on three bearing fault datasets and compares its diagnostic performance with that of representative PLL methods.

### 3.1. Dataset Description

Three bearing fault datasets, namely CWRU, MFPT, and PU, are used in the experiments. The classification tasks constructed from these datasets contain 10, 15, and 9 classes, respectively. The corresponding class definitions are summarized in Table 1. The selected data and sample construction procedures are described below.

#### 3.1.1. CWRU Dataset

**Figure 5:**
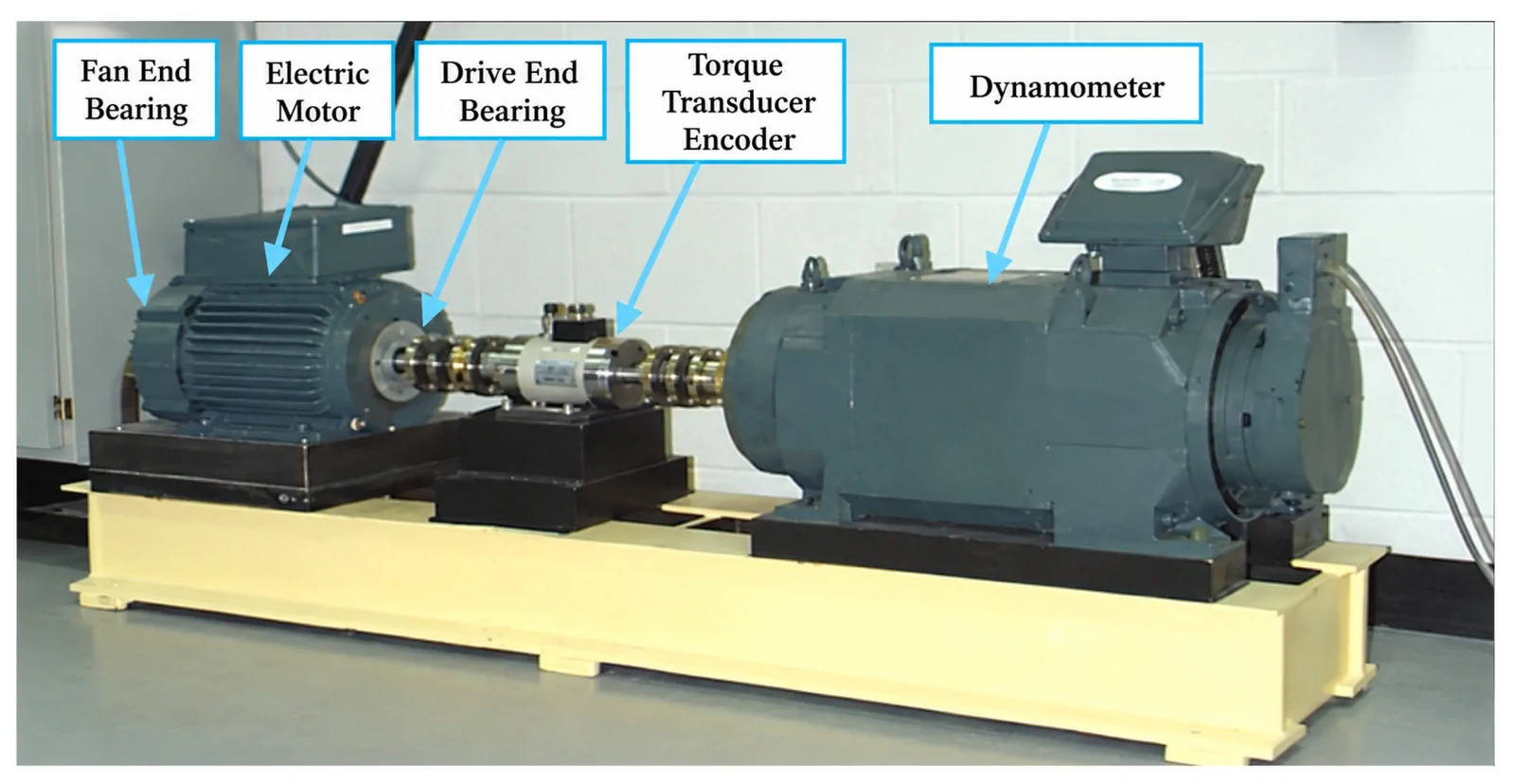
Bearing test rig for the CWRU dataset.

The Case Western Reserve University (CWRU) bearing dataset [38] contains vibration measurements collected from a bearing test rig, as shown in Figure 5. This study considers data obtained under a motor load of 3 hp and an approximate rotational speed of 1730 r/min, with a sampling frequency of 12 kHz. Ten classes are considered: one normal class and nine fault classes covering inner-race, outer-race, and ball faults, each with fault diameters of 0.007, 0.014, and 0.021 in. These classes are denoted by N, IR07–IR21, OR07–OR21, and B07–B21 in Table 1. To construct the samples, the vibration signals are segmented using a window length of 512 sampling points and a start-to-start stride of 1024 points. Each resulting segment is treated as an individual sample for subsequent preprocessing and classification.

#### 3.1.2. MFPT Dataset

**Figure 6:**
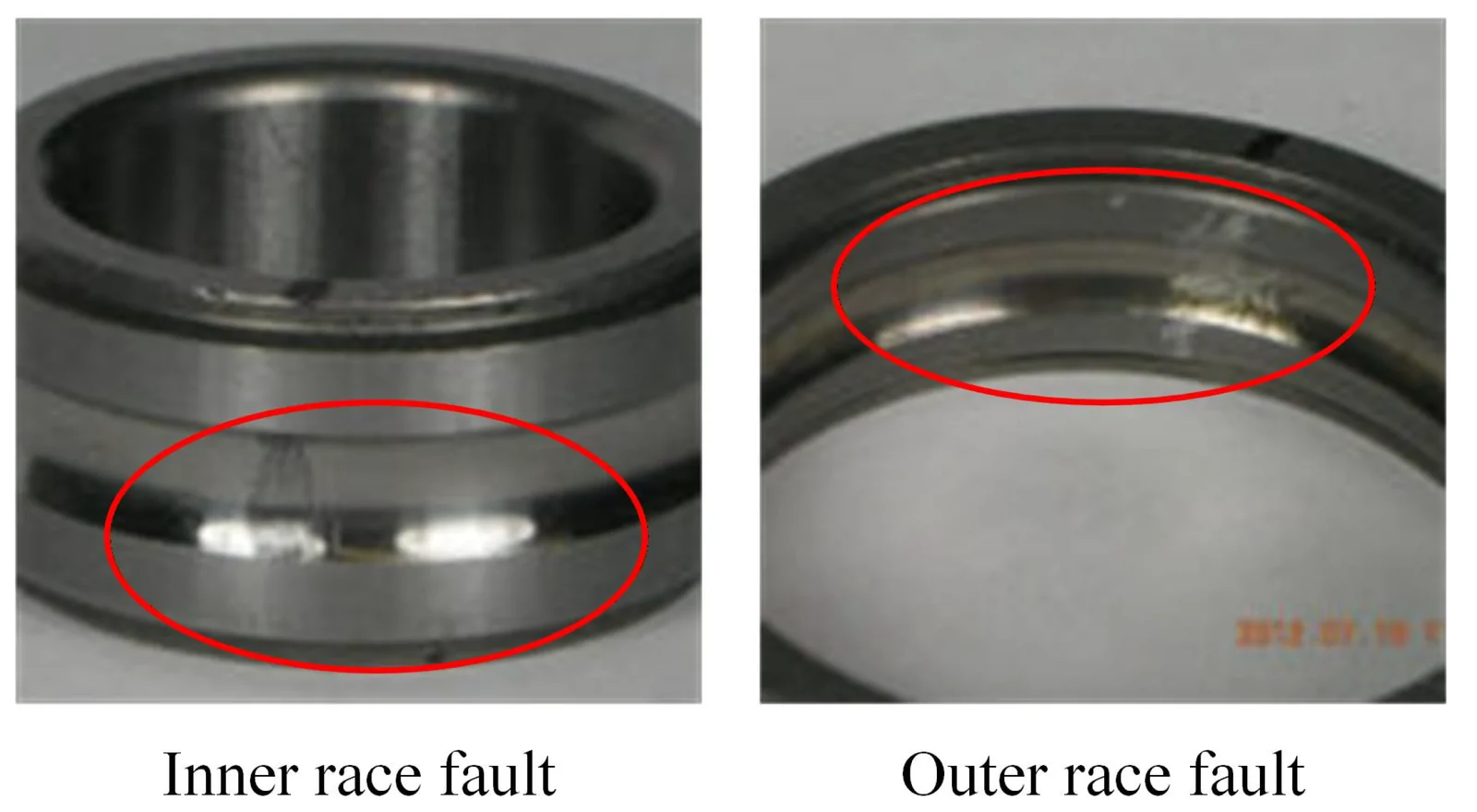
Illustration of bearing faults in the MFPT dataset.

The MFPT bearing dataset [39] is provided by the Society for Machinery Failure Prevention Technology. The selected test-rig data cover three bearing health conditions: normal, inner-race fault, and outer-race fault. Vibration signals were collected using an accelerometer mounted on the bearing housing. The bearing faults are illustrated in Figure 6. The normal-condition signals were recorded under a radial load of 270 lb at a sampling frequency of 97,656 Hz. The selected inner-race and outer-race fault signals were recorded at 48,828 Hz under seven radial load levels ranging from 0 to 300 lb and from 25 to 300 lb, respectively. In this study, each fault-type–load combination is treated as a separate class, resulting in a 15-class classification task comprising one normal class (N), seven inner-race fault classes (I1–I7), and seven outer-race fault classes (O1–O7). The normal-condition signals are resampled to 48,828 Hz to match the sampling frequency of the selected fault signals. All signals are then segmented using a window length of 2048 sampling points and a stride of 512 points, corresponding to a 75% overlap between adjacent windows.

#### 3.1.3. PU Dataset

**Figure 7:**
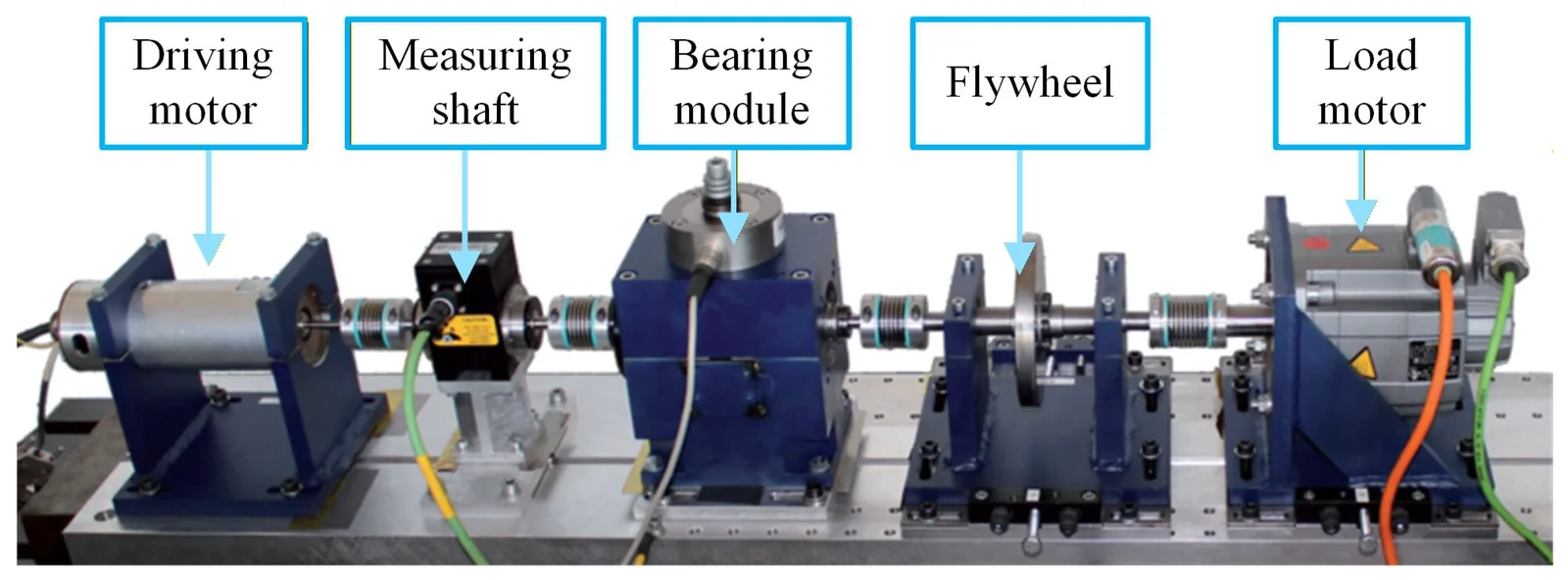
Bearing test rig for the PU dataset.

The Paderborn University (PU) bearing dataset [40] is used to evaluate the proposed method on bearings with damage generated through accelerated lifetime testing. The test rig, shown in Figure 7, consists of a driving motor, a measurement shaft, a bearing module, a flywheel, and a load motor. This study uses vibration data collected under operating condition N15_M07_F10, corresponding to a rotational speed of 1500 r/min, a load torque of 0.7 N·m, and a radial load of 1000 N. The vibration signals were measured at the bearing housing at a sampling frequency of 64 kHz. Nine bearing-specific classes are constructed using one healthy bearing, K001, and eight bearings with real damage: KA04, KA15, KA30, KB23, KB27, KI14, KI17, and KI21. Each selected bearing identifier corresponds to a separate class. The faulty bearings include outer-race damage, inner-race damage, and combined inner- and outer-race damage, covering different damage locations and characteristics. For sample construction, the vibration signals are segmented using a window length of 2048 sampling points and a start-to-start stride of 8192 points.

### 3.2. Data Processing and Experimental Setup

#### 3.2.1. Construction of Candidate Label Sets

**Figure 8:**
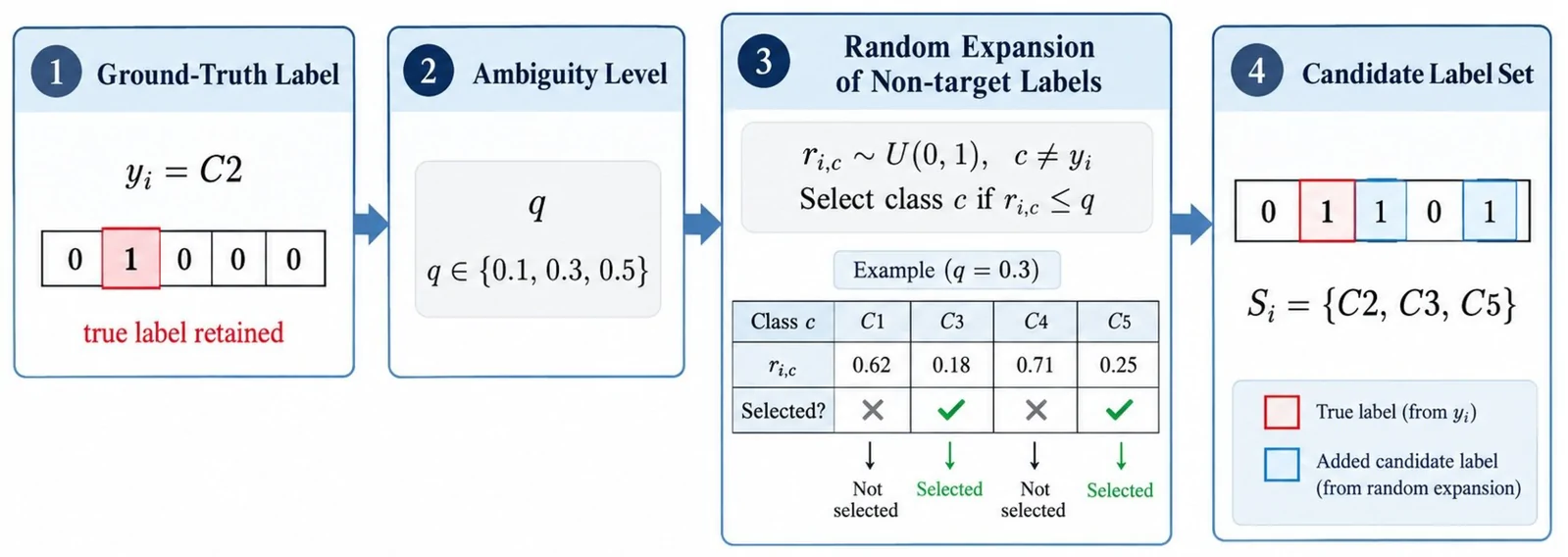
Construction of candidate label sets.

To evaluate the proposed method under different levels of label ambiguity, candidate label sets are synthetically generated from the original ground-truth annotations, as illustrated in Figure 8. Let $x_{i}$ denote the *i*-th sample with ground-truth label $y_{i}$, and let *K* denote the total number of classes. During candidate-label generation, the ground-truth label is always retained, while each non-target label is independently added with probability q∈[0,1]. Specifically, for each class c∈{1,…,K}, the binary candidate-label indicator is defined as

(16) $${\tilde{y}}_{i,c}=\left\{\begin{array}{cc} 1, & c=y_{i},\\ I(r_{i,c}\leq q), & c\neq y_{i}, \end{array}\right.\;r_{i,c}∼U(0,1),$$

where I(·) denotes the indicator function. The random variables $r_{i,c}$ are sampled independently for each sample and non-target class. The candidate label set $S_{i}$ consists of all classes for which ${\tilde{y}}_{i,c}=1$. This procedure guarantees that $y_{i}\in S_{i}$, while each non-target label is included with probability *q*.

Under this generation strategy, the expected size of the candidate label set is

(17) $$E\left[|S_{i}|\right]=1+\left(K-1\right)q.$$

A larger value of *q* therefore introduces more non-target labels on average, thereby increasing label ambiguity. In the experiments on the CWRU, MFPT, and PU datasets, *q* is set to 0.1, 0.3, and 0.5 to construct partial-label settings with different ambiguity levels. The same candidate-label generation protocol is used for all compared methods. Since the number of classes differs among datasets, the same value of *q* may result in different expected candidate-set sizes. Therefore, *q* is regarded as a candidate-label generation parameter rather than an identical ambiguity severity indicator across datasets. The comparisons are mainly conducted within each dataset under the same candidate-label generation protocol to ensure a fair evaluation. The expected candidate-set sizes for the three datasets under different ambiguity levels are summarized in Table 2.

#### 3.2.2. Implementation Details

To evaluate the robustness of the proposed method under noisy conditions, additive Gaussian noise is introduced into the vibration signals before time–frequency transformation. The noise intensity is controlled by the signal-to-noise ratio (SNR), defined as

(18) $$\mathrm{SNR}=10\log_{10}\left(\frac{P_{\mathrm{signal}}}{P_{\mathrm{noise}}}\right)$$

where $P_{\mathrm{signal}}$ and $P_{\mathrm{noise}}$ denote the power of the original vibration signal and the added Gaussian noise, respectively. Gaussian noise is independently generated for each vibration segment, with its power adjusted according to the corresponding clean signal to achieve the specified SNR. During both training and testing, noisy samples are generated at the corresponding SNR levels, and the same noise injection procedure is applied to all compared methods. In the noise robustness experiments, the SNR ranges from −2 to 6 dB. After noise injection, the one-dimensional vibration segments are transformed into two-dimensional time–frequency representations using the continuous wavelet transform (CWT). The resulting images are resized to 64×64 pixels and used as network inputs.

The vibration signals are first segmented into fixed-length samples using the sliding-window strategy. The generated samples are then randomly divided into training and test subsets at a ratio of 6:4 within each class, followed by continuous wavelet transform (CWT) to obtain time–frequency representations. The same data partitions are used for all compared methods to ensure consistent experimental conditions. However, this window-level splitting strategy may introduce correlations between training and test samples, particularly for the MFPT dataset with overlapping windows and the PU dataset with bearing-specific classes. Therefore, the evaluation results should be interpreted within the adopted experimental setting. Candidate label sets are generated for the training samples from their ground-truth annotations with the ambiguity parameter q∈{0.1,0.3,0.5}. A larger value of *q* increases the expected number of incorrect labels in each candidate set, thereby increasing the difficulty of label disambiguation. The same candidate-label generation protocol is applied to all compared methods to ensure a fair comparison.

The proposed method employs a modified ResNet-18 as the feature encoder, incorporating adaptive residual shrinkage to suppress noise-related responses and learn fault-discriminative representations. The embedding dimension is set to d=128. The learned representations are used by the classification branch and the ETF-guided prototype branch for joint partial-label learning. The temperature parameter of the prototype branch is set to $T_{\mathrm{proto}}=0.3$. RS-ETFPLL is trained for 500 epochs using stochastic gradient descent (SGD) with a batch size of 64 and an initial learning rate of 0.015. All experiments are implemented in PyTorch 2.5.1 under a Linux operating system and conducted on an NVIDIA GeForce RTX 3090 GPU. Each experimental setting is repeated ten times, and the classification results are reported as the mean accuracy (%) ± standard deviation over the ten runs.

#### 3.2.3. Comparison Methods

Six representative partial label learning methods are included for comparison. The selected baselines cover different technical paradigms of PLL, ranging from progressive label disambiguation and consistency-based risk optimization to contrastive representation learning and prototype-based modeling. This benchmark allows the proposed method to be evaluated from multiple perspectives, including candidate-label disambiguation, feature representation, and prototype learning. For a fair comparison, all methods employ ResNet-18-based feature extractors and the same CWT-based input representations. Identical data partitions and candidate-label generation protocols are adopted, and all methods are trained for 500 epochs. Specifically, the six baselines use standard ResNet-18 backbones, whereas RS-ETFPLL incorporates adaptive residual shrinkage blocks into the ResNet-18 architecture. The compared methods are summarized as follows.

- PRODEN [16]: A progressive label identification method that continuously updates the confidence of candidate labels according to the model predictions during training, thereby gradually reducing label ambiguity.
- CC [17]: A classifier-consistent PLL approach that learns the classification model directly from candidate-label supervision under a theoretically consistent learning formulation.
- RC [17]: A risk-consistent PLL method that reformulates the learning objective through a consistent risk estimator, providing a principled strategy for learning from ambiguous candidate labels.
- PiCO [18]: A contrastive partial label learning method that jointly performs representation learning and label disambiguation. Prototype information is exploited to refine candidate-label confidence and improve inter-class discrimination.
- ParSE [19]: A semantic representation-based PLL method that models the relationships between samples and labels in a shared representation space, enabling semantic information to assist candidate-label disambiguation.
- PaPi [20]: A PLL method in which a linear classifier guides a prototypical classifier sharing the same feature encoder, thereby improving visual representations under partial-label supervision.

### 3.3. Main Results

Table 3 presents the classification accuracies of RS-ETFPLL and six representative PLL baselines on the CWRU, MFPT, and PU datasets under three candidate-label ambiguity levels, q=0.1, 0.3, and 0.5. A larger value of *q* increases the probability of including incorrect labels in the candidate set. All methods are evaluated using the same data partitions and candidate-label generation protocol. The results are reported as mean classification accuracy ± standard deviation over repeated runs, with the highest mean accuracy in each setting highlighted in bold.

RS-ETFPLL achieves the highest mean classification accuracy in all nine experimental settings. At q=0.1, it obtains accuracies of 99.80%, 99.66%, and 99.21% on CWRU, MFPT, and PU, respectively, exceeding the strongest baseline in each setting by 0.30, 0.58, and 1.09 percentage points. At q=0.3, the corresponding accuracy improvements increase to 1.20, 1.99, and 1.57 percentage points. These results demonstrate the consistent performance advantage of RS-ETFPLL across the three datasets.

The advantage becomes more pronounced at the highest evaluated ambiguity level, q=0.5. Several baselines exhibit substantial performance degradation; for example, the accuracies of PRODEN and PiCO on PU decrease from 92.75% and 97.99% at q=0.1 to 66.98% and 69.88%, respectively. In contrast, RS-ETFPLL maintains accuracies of 99.59%, 98.99%, and 98.01% on CWRU, MFPT, and PU. The strongest competing methods in these three settings are PaPi, ParSE, and PaPi, respectively. Compared with these baselines, RS-ETFPLL improves the accuracy by 2.03, 4.46, and 2.16 percentage points. Thus, its margin over the strongest baseline increases with *q* on all three datasets, supporting its effectiveness under increasingly ambiguous candidate-label supervision.

As *q* increases from 0.1 to 0.5, the accuracy of RS-ETFPLL decreases by 0.21, 0.67, and 1.20 percentage points on CWRU, MFPT, and PU, respectively, while remaining above 98% in every evaluated setting. In addition, its standard deviations range from 0.02 to 0.09 percentage points across all nine settings, indicating low run-to-run variability under the adopted experimental protocol. Overall, these results demonstrate that RS-ETFPLL maintains high classification accuracy and stable performance across repeated runs, while exhibiting limited sensitivity to increasing candidate-label ambiguity within the evaluated range.

To further assess computational efficiency, Table 4 reports the number of trainable parameters, average training time per epoch, and inference time per batch for the compared methods. Compared with PaPi, RS-ETFPLL introduces only a slight increase in model parameters, with training and inference times increasing by 6.57% and 5.28%, respectively. Combined with the classification results in Table 3, these findings indicate that RS-ETFPLL achieves improved diagnostic accuracy with moderate additional computational overhead.

### 3.4. Noise Robustness Analysis

To evaluate the noise robustness of RS-ETFPLL, experiments are conducted with the candidate-label ambiguity parameter fixed at q=0.5 and the SNR set to −2, 0, 2, 4, and 6 dB. The proposed method is compared with six representative PLL methods on the CWRU, MFPT, and PU datasets. Table 5 reports the mean classification accuracy and standard deviation, with the highest mean accuracy in each setting highlighted in bold.

RS-ETFPLL achieves the highest mean classification accuracy across all three datasets and all five SNR levels. At the lowest evaluated SNR of −2 dB, it obtains accuracies of 81.72%, 82.08%, and 73.78% on CWRU, MFPT, and PU, respectively. These results exceed those of the best-performing baseline on each dataset by 10.13, 9.77, and 13.44 percentage points. At 0 dB, the corresponding gains are 8.78, 8.42, and 10.24 percentage points. These comparisons demonstrate the advantage of RS-ETFPLL when noise interference and candidate-label ambiguity coexist.

As the SNR increases, the mean accuracy of every method improves on each dataset, while RS-ETFPLL maintains its leading performance. At 6 dB, it outperforms the best-performing baselines by 3.33, 4.58, and 7.37 percentage points on CWRU, MFPT, and PU, respectively. The performance gaps are larger at lower SNRs, indicating a more pronounced advantage under stronger noise interference. In addition, the standard deviations of RS-ETFPLL range from 0.07 to 0.36 percentage points. Overall, these results support the effectiveness of RS-ETFPLL under the evaluated noisy and ambiguous supervision settings. These results are obtained under matched Gaussian noise conditions, while generalization to unseen noise levels and non-Gaussian disturbances remains to be investigated.

### 3.5. Feature Visualization Analysis

**Figure 9:**
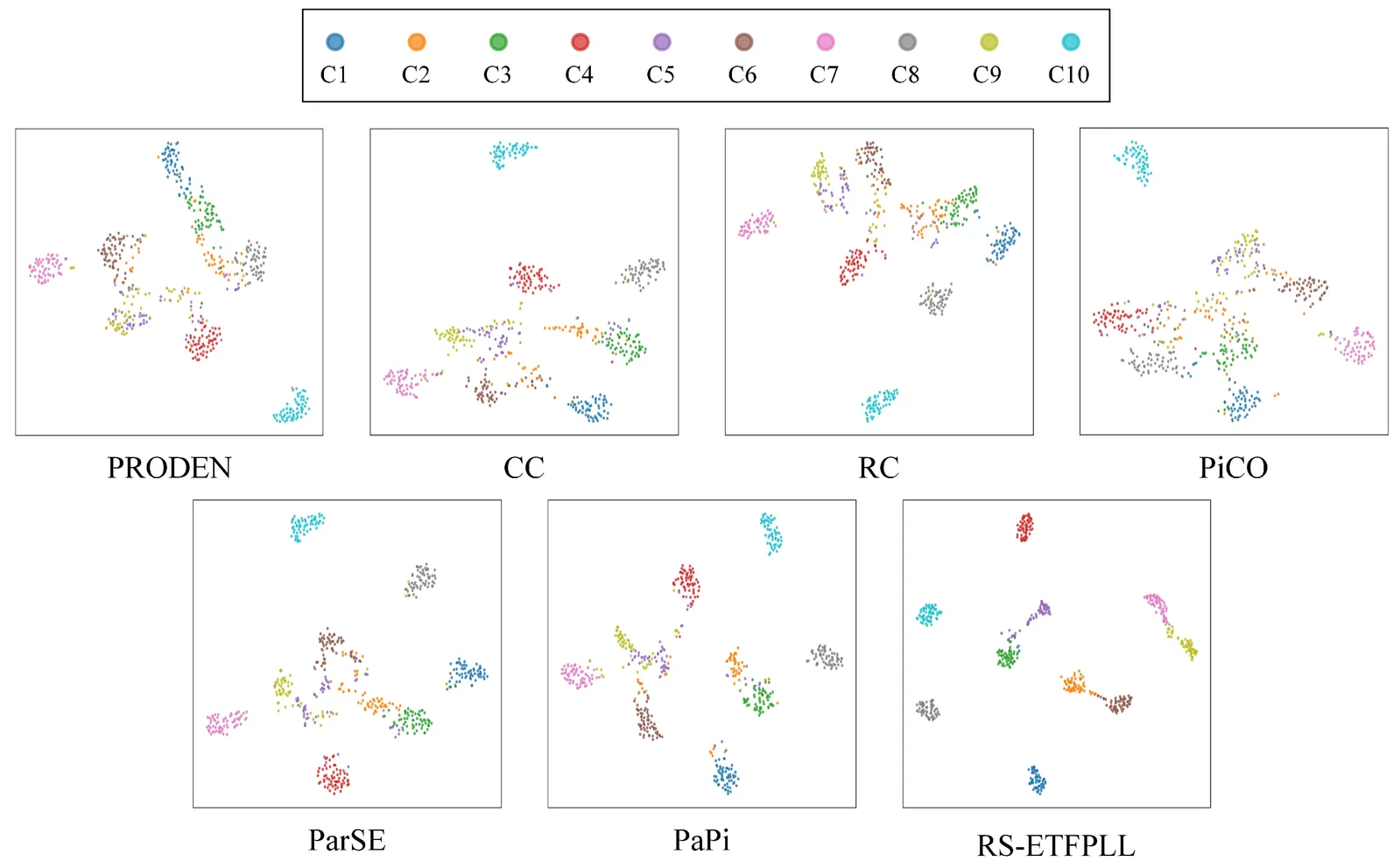
t-SNE visualization of different methods on the CWRU dataset at q=0.5 and SNR = 0 dB.

**Figure 10:**
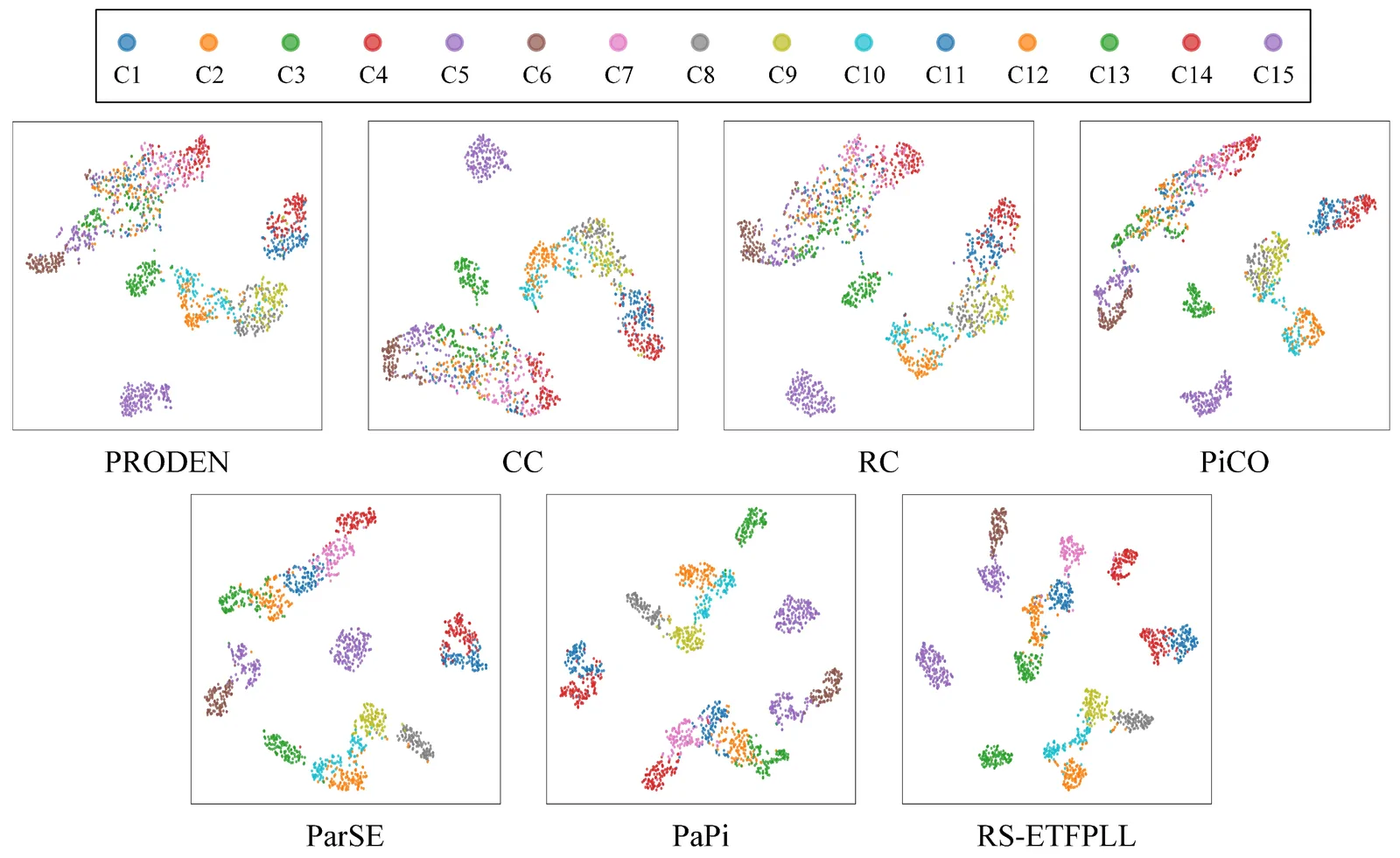
t-SNE visualization of different methods on the MFPT dataset at q=0.5 and SNR = 2 dB.

**Figure 11:**
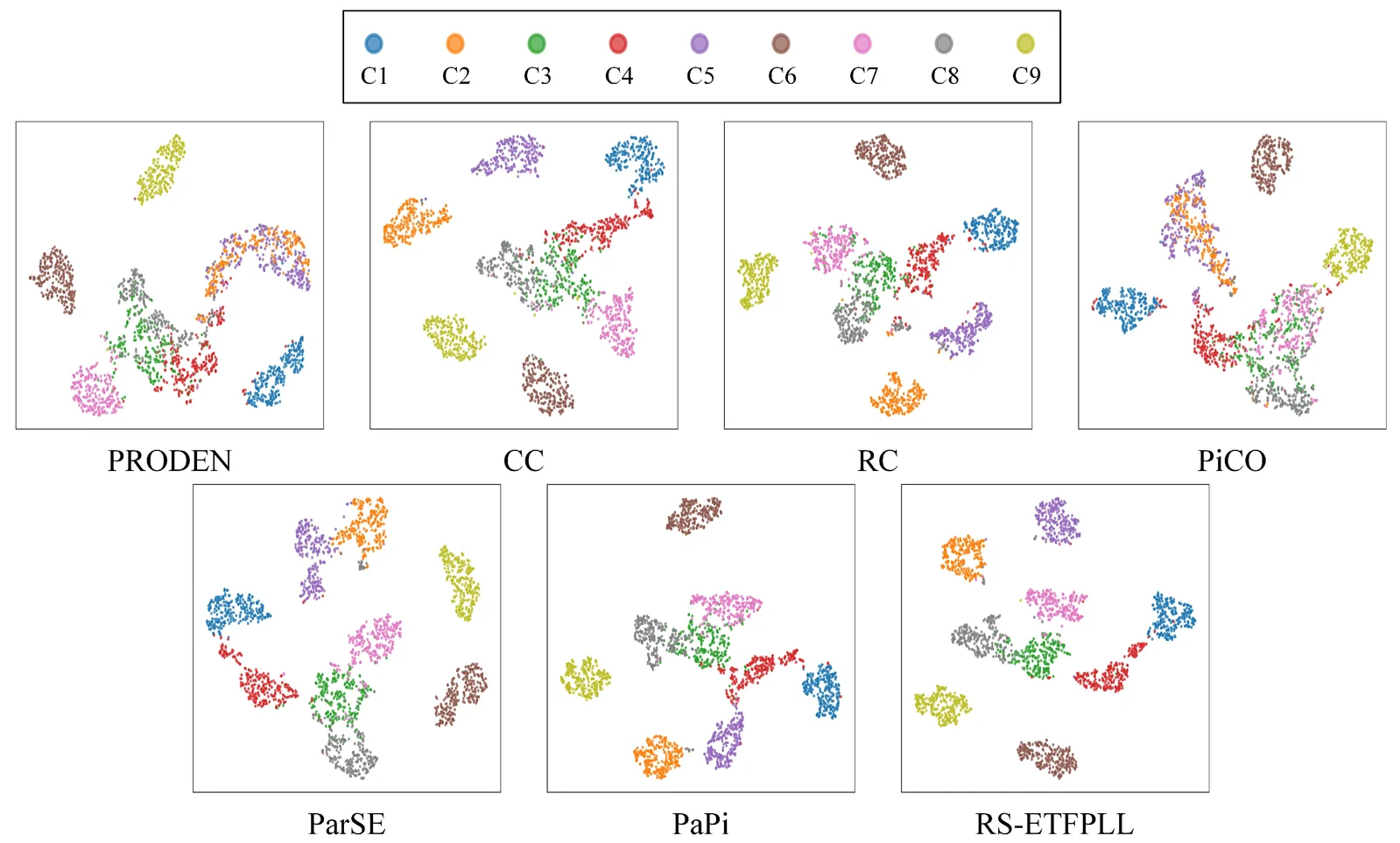
t-SNE visualization of different methods on the PU dataset at q=0.5 and SNR = 4 dB.

To further investigate the discriminability of the learned feature representations, t-SNE is employed to project the high-dimensional features into a two-dimensional space for visualization. With the candidate-label ambiguity level fixed at q=0.5, representative noise conditions of 0 dB, 2 dB, and 4 dB are selected for the CWRU, MFPT, and PU datasets, respectively. Figure 9, Figure 10 and Figure 11 show the resulting feature distributions, where different colors denote different classes. In addition, the Silhouette Score is used to quantitatively evaluate the clustering structure, where a higher value indicates better intra-class compactness and inter-class separability. The corresponding results are reported in Table 6.

As shown in Figure 9, Figure 10 and Figure 11, several competing methods exhibit noticeable class overlap or relatively scattered feature distributions, especially in the central regions of the projected feature space. Although ParSE and PaPi improve class separation to some extent, neighboring clusters are still partially mixed on some datasets. In contrast, RS-ETFPLL consistently produces more compact and better-separated feature clusters across all three datasets. This visual observation is further supported by the Silhouette Scores in Table 6. Specifically, RS-ETFPLL achieves the highest scores of 0.5855, 0.3797, and 0.4851 on CWRU, MFPT, and PU, respectively, compared with the best baseline scores of 0.4048, 0.3008, and 0.4098, all achieved by PaPi.

Overall, both the visualized feature distributions and the quantitative clustering metric indicate that RS-ETFPLL learns a more discriminative feature space than the competing PLL methods. The results suggest that the overall design of RS-ETFPLL is beneficial for learning feature representations with improved intra-class compactness and inter-class separation, which is consistent with its superior classification performance under noisy and ambiguous supervision.

### 3.6. Ablation Study

To assess the contributions of the RS module and the ETF-guided prototype representation, component ablation experiments are conducted on the CWRU, MFPT, and PU datasets at SNRs of 0 and 2 dB, with the candidate-label ambiguity level fixed at q=0.5. Four variants are evaluated: a baseline without either component, two single-component variants incorporating only RS or only ETF, and the complete model incorporating both. All remaining training settings are kept unchanged. The classification results are reported in Table 7. As shown in Table 7, incorporating either component alone improves classification accuracy over the baseline. At 0 dB, adding RS alone yields improvements of 1.57, 1.56, and 1.66 percentage points on CWRU, MFPT, and PU, respectively. Adding ETF alone provides corresponding gains of 2.32, 2.35, and 2.77 percentage points. When both components are included, the accuracies increase to 92.53%, 89.12%, and 79.86%, exceeding the baseline by 3.79, 5.21, and 5.24 percentage points, respectively.

A similar trend is observed at 2 dB, where both single-component variants outperform the baseline and the complete model achieves the highest accuracy on each dataset. Specifically, the complete model obtains accuracies of 95.96%, 93.52%, and 87.11% on CWRU, MFPT, and PU, corresponding to improvements of 2.39, 4.20, and 3.58 percentage points over the baseline. The gains of the complete model over the baseline are larger at 0 dB than at 2 dB on all three datasets, suggesting greater benefits under the more severe of the two evaluated noise conditions.

Importantly, the complete model outperforms both single-component variants across all six dataset–SNR settings. Relative to the ETF-only variant, which is the stronger single-component model in every setting, the complete model provides additional gains of 1.47, 2.86, and 2.47 percentage points at 0 dB, and 1.11, 2.18, and 1.39 percentage points at 2 dB on CWRU, MFPT, and PU, respectively. These comparisons show that combining RS and ETF provides additional benefits beyond using either component alone, supporting their complementary contributions to classification under the evaluated noisy partial-label settings. Further comparisons with alternative prototype geometries could provide additional insights into the specific contribution of the equiangular structure.

### 3.7. Structured Candidate-Label Ambiguity Analysis

To further evaluate RS-ETFPLL under structured label ambiguity, a similarity-aware candidate-label generation protocol is introduced. Specifically, incorrect labels belonging to the same fault group are included with a probability of 0.7, while other incorrect labels are included with a probability of 0.5. The fault groups are defined as B, IR, and OR for CWRU; I and O for MFPT; and KA, KB, and KI for PU. The ground-truth label is always retained, while healthy samples contain only the healthy label.

**Figure 12:**
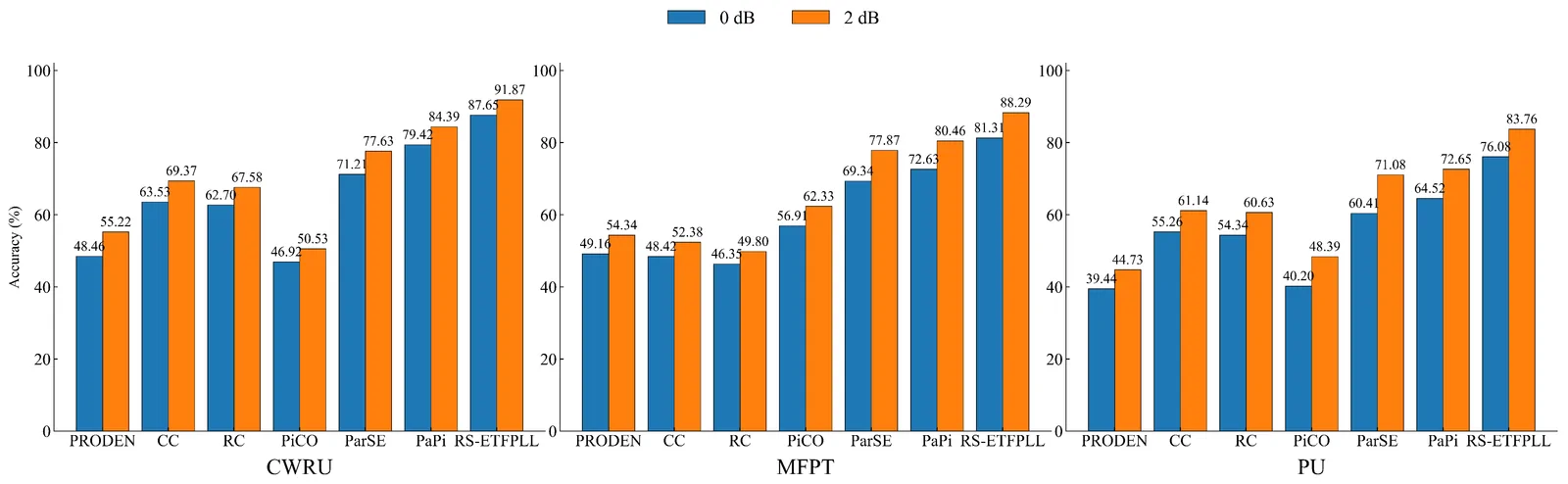
Classification accuracy of different PLL methods under similarity-aware candidate-label ambiguity on the CWRU, MFPT, and PU datasets at SNRs of 0 and 2 dB.

Experiments are conducted on the three datasets at SNRs of 0 and 2 dB, and the results are presented in Figure 12. As shown in the figure, RS-ETFPLL achieves the highest classification accuracy across all six settings. Compared with the strongest baseline, PaPi, the proposed method achieves improvements of 8.23 and 7.48 percentage points on CWRU, 8.68 and 7.83 percentage points on MFPT, and 11.56 and 11.11 percentage points on PU at 0 and 2 dB, respectively. These results indicate that RS-ETFPLL maintains its performance advantage under the evaluated similarity-aware candidate-label ambiguity conditions.

## 4. Conclusions

This paper proposes RS-ETFPLL to address noise interference and candidate-label ambiguity in bearing fault diagnosis. The framework integrates adaptive residual shrinkage for noise-robust feature extraction with fixed simplex ETF prototypes that define a structured and uniformly separated class geometry. The classification and prototype branches are jointly optimized using candidate-label supervision, while pseudo-soft labels obtained by normalizing classification predictions within the candidate sets further guide prototype learning. This design facilitates label disambiguation and discriminative representation learning.

Experiments on the CWRU, MFPT, and PU datasets show that RS-ETFPLL achieves the highest mean classification accuracy across all evaluated candidate-label ambiguity levels. In the main comparison at q=0.5, it outperforms the strongest baseline on each dataset by 2.03, 4.46, and 2.16 percentage points, respectively. The noise experiments further support its effectiveness under the evaluated SNR conditions. Additional experiments under similarity-aware candidate-label ambiguity further demonstrate the effectiveness of RS-ETFPLL under structured label uncertainty. In addition, the t-SNE visualizations and silhouette scores provide supporting evidence of improved class clustering, while the component ablations support the effectiveness and complementary contributions of residual shrinkage and the ETF-guided prototype representation.

Overall, the results indicate that combining noise-robust feature learning with structured class representations is effective for partial-label bearing fault diagnosis under the tested conditions. In practical applications, RS-ETFPLL could potentially be integrated into an intelligent bearing condition monitoring system, where vibration signals are transformed into time–frequency representations for subsequent fault prediction. However, the current study relies on public bearing datasets, with candidate-label ambiguity simulated through random and similarity-aware generation protocols and noise introduced through additive Gaussian noise. Future work will investigate realistic instance-dependent partial-label learning, non-Gaussian and time-varying noise, class imbalance, and cross-condition distribution shifts to further assess the generalization of RS-ETFPLL in practical machinery health monitoring applications.

## Figures and Tables

**Table 1:** Class definitions of the three datasets used in this study.

Class	CWRU	MFPT	PU
C1	N	N	K001
C2	IR07	I1	KA04
C3	IR14	I2	KA15
C4	IR21	I3	KA30
C5	OR07	I4	KB23
C6	OR14	I5	KB27
C7	OR21	I6	KI14
C8	B07	I7	KI17
C9	B14	O1	KI21
C10	B21	O2	–
C11	–	O3	–
C12	–	O4	–
C13	–	O5	–
C14	–	O6	–
C15	–	O7	–

**Table 2:** Expected candidate-set sizes of the three datasets under different ambiguity levels.

Dataset	q=0.1	q=0.3	q=0.5
CWRU	1.9	3.7	5.5
MFPT	2.4	5.2	8.0
PU	1.8	3.4	5.0

**Table 3:** Classification accuracy (%) of different PLL methods on the CWRU, MFPT, and PU datasets under different ambiguity levels. The results are reported as mean ± standard deviation, and the best result in each setting is highlighted in bold.

Dataset	Method	q=0.1	q=0.3	q=0.5
CWRU	PRODEN	95.81±0.26	87.16±0.42	79.50±1.29
	CC	98.01±0.17	96.59±0.24	91.89±0.50
	RC	97.99±0.24	96.53±0.28	93.14±0.72
	PiCO	97.11±0.33	94.11±0.32	73.78±0.99
	ParSE	97.35±0.36	95.39±0.39	92.47±0.43
	PaPi	99.50±0.08	98.52±0.10	97.56±0.18
	RS-ETFPLL	99.80±0.02	99.72±0.04	99.59±0.05
MFPT	PRODEN	94.50±0.61	93.26±0.70	85.53±0.90
	CC	94.69±0.10	93.49±0.22	82.89±0.88
	RC	94.01±0.15	92.07±0.35	79.63±0.77
	PiCO	96.82±0.11	96.59±0.17	81.67±0.23
	ParSE	97.27±0.19	96.45±0.28	94.53±0.38
	PaPi	99.08±0.05	97.61±0.17	93.14±0.27
	RS-ETFPLL	99.66±0.05	99.60±0.07	98.99±0.08
PU	PRODEN	92.75±0.68	88.13±0.82	66.98±0.85
	CC	95.70±0.57	94.35±0.55	89.38±0.98
	RC	96.70±0.52	95.36±0.32	90.46±0.75
	PiCO	97.99±0.15	94.78±0.34	69.88±0.44
	ParSE	94.69±0.24	93.53±0.38	91.68±0.35
	PaPi	98.12±0.05	97.28±0.10	95.85±0.09
	RS-ETFPLL	99.21±0.03	98.85±0.07	98.01±0.09

**Table 4:** Computational complexity comparison of different PLL methods.

Method	Params (M)	Training (s/Epoch)	Inference (ms/Batch)
PRODEN	11.49	20.26	9.05
CC	11.48	19.78	8.30
RC	11.48	19.55	8.26
PiCO	11.50	26.62	11.89
ParSE	11.50	24.55	11.45
PaPi	11.50	27.55	12.31
RS-ETFPLL	11.59	29.36	12.96

**Table 5:** Classification accuracy (%) of different methods on the CWRU, MFPT, and PU datasets under different SNR levels with q=0.5. Bold values indicate the best classification accuracy under each experimental setting.

Dataset	Method	−2 dB	0 dB	2 dB	4 dB	6 dB
CWRU	PRODEN	50.81±1.41	59.09±1.23	65.75±1.08	72.84±0.96	75.66±0.88
	CC	55.36±0.82	71.87±0.69	75.05±0.61	80.42±0.56	84.32±0.51
	RC	53.54±0.89	69.56±0.76	74.29±0.68	80.56±0.61	85.25±0.55
	PiCO	48.27±1.16	55.39±1.03	58.14±0.95	62.11±0.88	65.32±0.81
	ParSE	65.24±0.71	77.43±0.60	83.32±0.53	87.89±0.48	89.12±0.44
	PaPi	71.59±0.42	83.75±0.35	90.02±0.28	93.65±0.24	95.88±0.20
	RS-ETFPLL	81.72±0.36	92.53±0.28	95.96±0.20	98.28±0.11	99.21±0.07
MFPT	PRODEN	52.31±1.08	58.96±0.97	64.23±0.89	73.65±0.78	80.22±0.69
	CC	48.88±1.02	56.25±0.91	60.01±0.84	70.64±0.76	76.55±0.68
	RC	43.23±0.95	53.22±0.87	56.36±0.81	67.46±0.73	71.67±0.66
	PiCO	54.26±0.54	64.78±0.46	71.26±0.39	77.28±0.33	81.91±0.29
	ParSE	70.55±0.63	79.20±0.54	86.98±0.46	90.56±0.40	92.01±0.36
	PaPi	72.31±0.44	80.70±0.38	87.66±0.32	90.23±0.29	91.55±0.25
	RS-ETFPLL	82.08±0.22	89.12±0.17	93.52±0.17	95.87±0.15	96.59±0.12
PU	PRODEN	36.66±1.02	41.91±0.94	48.23±0.88	55.56±0.81	59.20±0.74
	CC	52.13±1.11	59.62±1.03	67.99±0.95	71.05±0.88	75.33±0.80
	RC	54.88±0.90	60.35±0.83	68.02±0.77	72.30±0.71	78.65±0.65
	PiCO	39.22±0.62	43.22±0.57	51.26±0.52	58.54±0.49	62.33±0.45
	ParSE	58.21±0.58	66.35±0.50	76.42±0.43	80.06±0.38	82.45±0.35
	PaPi	60.34±0.21	69.62±0.18	78.96±0.15	82.78±0.12	85.21±0.10
	RS-ETFPLL	73.78±0.34	79.86±0.22	87.11±0.16	90.71±0.08	92.58±0.08

**Table 6:** Silhouette scores of different methods on three datasets under the selected SNR conditions with q=0.5. Bold values indicate the highest silhouette score for each dataset.

Method	CWRU/0 dB	MFPT/2 dB	PU/4 dB
PRODEN	0.2646	0.1902	0.2121
CC	0.3331	0.1875	0.3392
RC	0.3140	0.1777	0.3775
PiCO	0.2364	0.2354	0.2281
ParSE	0.3636	0.2995	0.3760
PaPi	0.4048	0.3008	0.4098
RS-ETFPLL	**0.5855**	**0.3797**	**0.4851**

**Table 7:** Ablation results of the RS module and ETF-guided prototype representation under different SNR levels with q=0.5. ✓ and × indicate whether the corresponding module is enabled or disabled, respectively. Bold values indicate the best classification accuracy under each experimental setting.

SNR	RS	ETF	CWRU	MFPT	PU
0 dB	×	×	88.74	83.91	74.62
	×	✓	91.06	86.26	77.39
	✓	×	90.31	85.47	76.28
	✓	✓	**92.53**	**89.12**	**79.86**
2 dB	×	×	93.57	89.32	83.53
	×	✓	94.85	91.34	85.72
	✓	×	94.62	90.93	84.88
	✓	✓	**95.96**	**93.52**	**87.11**

## Data Availability

Publicly available datasets were analyzed in this study.
